# Transcriptomic Hallmarks of Ischemia-Reperfusion Injury

**DOI:** 10.3390/cells10071838

**Published:** 2021-07-20

**Authors:** Mandana Movahed, Sydney Brockie, James Hong, Michael G. Fehlings

**Affiliations:** 1 Division of Genetics and Development, Krembil Research Institute, University Health Network, Toronto, ON M5T 2S8, Canada; mandana.movahed@mail.utoronto.ca (M.M.); s.brockie@mail.utoronto.ca (S.B.); jms.hong@mail.utoronto.ca (J.H.); 2 Institute of Medical Science, University of Toronto, Toronto, ON M5S 1A8, Canada; 3 Spinal Program, University Health Network, Toronto Western Hospital, Toronto, ON M5T 2S8, Canada

**Keywords:** ischemia reperfusion injury, RNA-seq, transcriptomics

## Abstract

Ischemia reperfusion injury (IRI) is associated with a broad array of life-threatening medical conditions including myocardial infarct, cerebral stroke, and organ transplant. Although the pathobiology and clinical manifestations of IRI are well reviewed by previous publications, IRI-related transcriptomic alterations are less studied. This study aimed to reveal a transcriptomic hallmark for IRI by using the RNA-sequencing data provided by several studies on non-human preclinical experimental models. In this regard, we focused on the transcriptional responses of IRI in an acute time-point up to 48 h. We compiled a list of highly reported genes in the current literature that are affected in the context of IRI. We conducted Gene Ontology (GO) and Kyoto Encyclopedia of Genes and Genomes (KEGG) enrichment analyses and found many of the up-regulated genes to be involved in cell survival, cell surface signaling, response to oxidative stress, and inflammatory response, while down-regulated genes were predominantly involved in ion transport. Furthermore, by GO analysis, we found that multiple inflammatory and stress response processes were affected after IRI. Tumor necrosis factor alpha (TNF) and nuclear factor kappa-light-chain-enhancer of activated B cells (NF-κB) signaling pathways were also highlighted in the Kyoto Encyclopedia of Genes and Genomes enrichment analysis. In the last section, we discuss the treatment approaches and their efficacy for IRI by comparing RNA sequencing data from therapeutic interventions with the results of our cross-comparison of differentially expressed genes and pathways across IRI.

## 1. Introduction

Ischemia reperfusion injury (IRI) is imposed by the transient loss of circulation to a specific tissue for various durations of time, followed by the restoration of blood flow that results in widespread inflammatory responses causing secondary injury [1]. IRI commonly occurs in myocardial infarction [2], cerebral stroke [3], and cases of transplantation of the kidney [4] and liver [5]. Across these injury modalities, loss of circulation causes ischemic hypoxia, which has vast effects in cell metabolism, ionic balance maintenance, and mitochondrial function [1].

In hypoxic conditions, glycolysis becomes the sole energy-producing process and the mitochondrial electron transport system is inhibited, resulting in buildup of glycolytic by-products and reactive oxidative species (ROS) [6]. This disrupts ionic balances, thereby disrupting transmembrane potential. In the mitochondria, a lack of oxygen causing buildup of hydrogen ions across the membrane may cause the mitochondrial permeability transition pore to open, decreasing transmembrane potential and ultimately resulting in bursting and subsequent release of apoptogens and organelle components, contributing to damage-associated molecular patterns [6]. Similarly, buildup of ROS caused by mitochondrial dysfunction impairs protein folding in the endoplasmic reticulum [7] and activate protein complexes, such as mitogen-activated protein kinase (MAPK) [8], calcium/calmodulin-dependent protein kinase (CaMK) [9], protein kinase C [10], and receptor-interacting protein kinases [11], which initiate several pro-inflammatory cascades. Ionic imbalances inflict similar damage across the cell membrane, where voltage-gated channels respond to the absence of oxygen by increasing ionic exchange to maintain tonicity, eventually leading to excess intracellular calcium and cytotoxicity [12]. In cases of stroke, the dramatic release of glutamate from calcium-saturated cells causes widespread neuronal glutamatergic excitotoxicity and cell death, and inhibiting sodium channels with was found to attenuate this cascade [13].

Ischemia and ischemia reperfusion effects were also observed on the genomic level, where ROS were found to drive epigenetic changes in expression of mRNA [14] and noncoding RNAs, facilitating an array of further cellular responses [1]. While the molecular footprint of IRI was extensively studied and articulated, transcriptional responses have only recently garnered attention and lack any in-depth characterization. Moreover, these transcriptional events are likely to play a far more significant role than is currently appreciated, and understanding them is critical to addressing the pathophysiology of IRI [14]. Here, we will review several recent studies in order to reveal a transcriptomic hallmark for IRI during acute time-point. We will do this by comparing the differential expression of genes reported after the onset of IRI. Table 1 presents a summary of the articles that are used in the paper.

## 2. Methods

### 2.1. Systematic Review

The PubMed database (https://pubmed.ncbi.nlm.nih.gov/, accessed on 14 April 2021) was thoroughly searched from inception to April 2021. Three searches were conducted using the following key words in either the abstract or the title: (“IRI” AND “RNA-sequencing”), (“ischemia reperfusion injury” AND “RNA-sequencing”), and (“ischemia reperfusion injury” AND “transcriptomic”). The selected articles had to contain open access datasets with the full list of differentially expressed genes (DEGs) from bulk RNA-sequencing. Studies on single-cell RNA-sequencing were excluded. The changes in the mRNA profile were extracted for the acute phase (from the injury onset up to 48 h) post-IRI. Selected articles also had to contain either naïve control or uninjured sham in order to compare the normal gene expression profile and the IRI-related changes in the mRNA profile. The studies reporting gene expression changes only after applying a treatment for IRI were excluded.

### 2.2. Data Extraction and Handling

We extracted the DEGs (adjusted *p*-value < 0.05) from each publication and cross-referenced them based on the reported gene symbols. The resultant list included genes reported with varying frequencies (scores) across studies ranging from 7 to 1. Next, this list was filtered for consistency. We kept only the genes that had consistent expression directionality in at least 75% of the reports for that gene. We then divided these genes into up- and down-regulated lists. Finally, we ranked these genes based on the number of times they were reported across studies. The genes with 4 or more reports (out of 7) were identified as “commonly reported DEGs” (Tables S4 and S5), which were used for further enrichment analysis. The highest ranked items of up- and down-regulated lists were represented as “highly reported DEGs” in Table 2 and Table 3. All the initial data handling was performed on Excel version 2016 (Microsoft; WA, USA).

### 2.3. GGO Enrichment Analysis

The GO categories “Molecular Function (MF)”, “Biological Process (BP)”, and “Cellular Components (CC)” were analyzed for significantly enriched terms using the PANTHER enrichment test [32]. The analysis was run against the Mus musculus reference list using Fisher’s exact test and p-values were corrected by false discovery rate (FDR) calculation. Finally, all terms with an FDR < 0.05 were collected and the top 20 terms for each category were graphed using the ggplot2 package in R [33].

### 2.4. KEGG Analysis

The most highly affected KEGG pathways were isolated using the clusterProfiler package in R [33,34]. Commonly repeated DEGs were enriched with KEGG dataset for the Mus musculus organism (organism code: mmu) and pathways with an adjusted *p*-value of < 0.05 were plotted. Finally, the top 20 significant KEGG pathways were graphed on bubble charts using the ggplot2 package in R.

### 2.5. Venn Diagram

We graphed the DEG lists from all studied publications (genes with adjusted *p*-values of <0.05) using the Venn diagrams drawer online tool (http://bioinformatics.psb.ugent.be/webtools/Venn/, accessed on 20 June 2021). To analyze organ-specific changes, we combined the DEG lists from studies on cerebral models (three publications) and used this in our comparison across organ models (spinal cord, hepatic, renal, and myocardial; one publication per organ).

## 3. Results

### 3.1. Transcriptomic Hallmark of Ischemic Reperfusion Injury

The injurious effects of reperfusion injury appear over hours and days following ischemia. In this article, we first aimed to outline the transcriptomic hallmark of IRI occurring over the 48-h window post-ischemia. We did this by conducting a comparison analysis between the DEGs of mRNA sequencing data provided by multiple studies on non-human preclinical experimental models (full list is provided in Table S1). In our comparative analysis, we only considered the genes that were consistently reported to be either up- or down-regulated. The genes with the highest consistency score are presented as “highly repeated DEGs” in Table 2 and Table 3. Among the upregulated genes, there are multiple inflammatory response genes, including *Bcl3*, *Tnfrsf1a*, *Cxcl1*, and *Tlr2*. Furthermore, the upregulation of *Litaf*, *Myc*, *Stat3*, *Atf3*, and *Cyr61* suggested the involvement of cell survival and apoptotic pathways. Other contributing processes may be cell surface adhesion and signaling (*Icam1*, *Spon2*, and *Itga5*) and oxidative stress response (*Hspb1* and *Hmox1*). Interestingly, most of the consistently downregulated genes related to ion channels (e.g., *Scn4b*, *Cabp1*, *Kcnab1*, *Kcnt1*, *Kcng1*, and *Cacnb3*). Other downregulated genes play roles in cytoskeleton regulation (*Pclo* and *Cobl*) and response to hypoxia and DNA damage (*Dlg2*).

Although the selected articles for comparative analysis performed bulk RNA sequencing, we noticed several of our highly repeated DEGs matched with single-cell RNA sequencing results from Zamanian et al., 2012. In this study, the authors isolated astrocytes from an ischemic condition, and reported differential expression patterns [35]. The results suggested that astrocytes from the ischemic condition displayed a protective phenotype with a specific gene expression pattern from other types of injuries, such as neuroinflammation. Notably, some of our highly repeated DEGs were similar to the reported genes for these protective astrocytes from the ischemic condition (*Litaf*, *Bcl3*, *Hmox1*, *Myc*, *Stat3*, *Atf3*, *Icam1*, *Tnfrsf1a*, *Cxcl1*, and *Hspb1*). This information supports the notion that astrocytes may be one of the key contributors to IRI and likely play a protective role in this context.

### 3.2. Pathways Involved in Ischemia Reperfusion Injury

**Figure 1:**
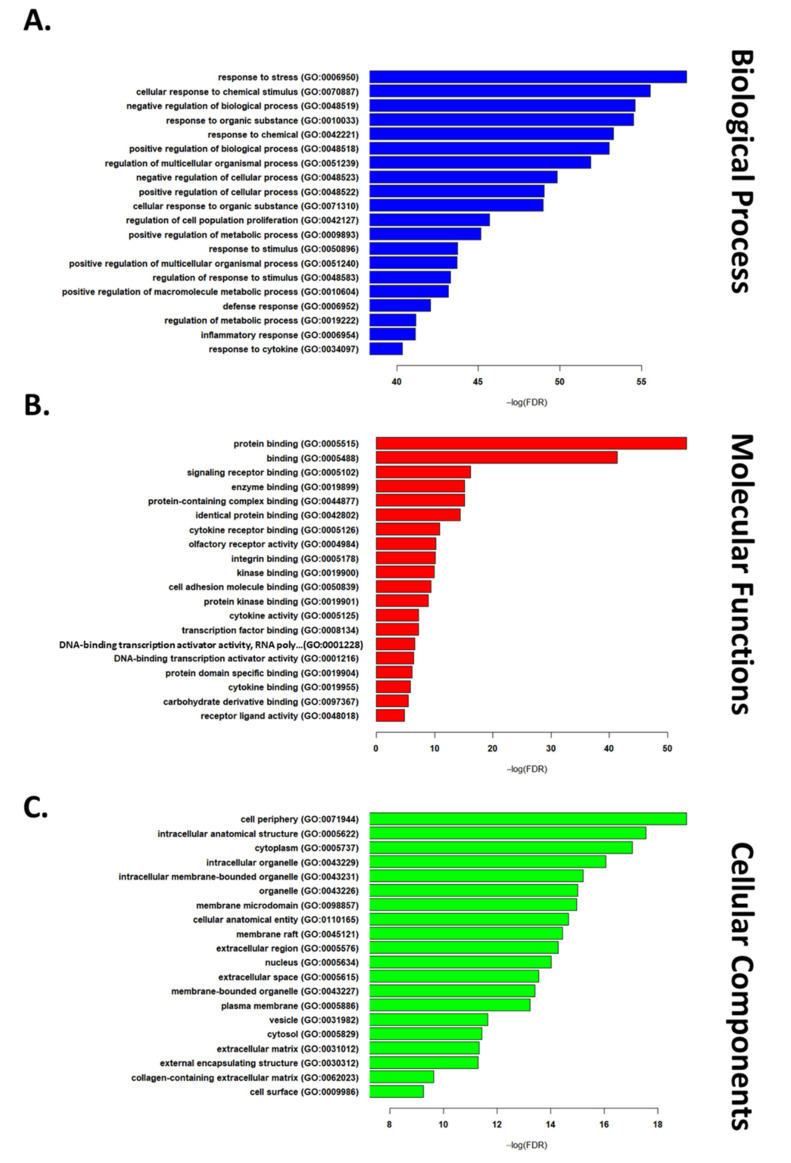
The top 20 GO terms in each of the biological process (**A**), cellular components (**B**), and molecular functions (**C**) domains for upregulated genes (list of all significant terms can be found in Tables S6–S8).

**Figure 2:**
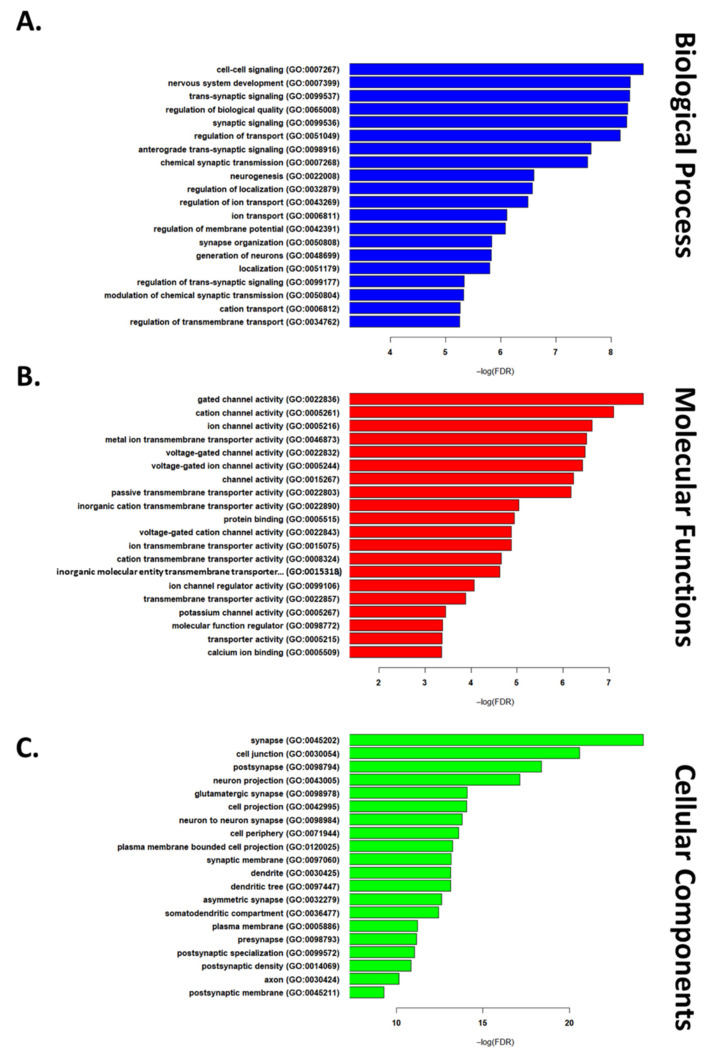
The top 20 GO terms in each of the biological process (**A**), cellular components (**B**), and molecular functions (**C**) domains for downregulated genes (list of all significant terms can be found in Tables S9–S11).

Next, we used a list of “commonly reported DEGs” in IRI conditions (4 out of 7) to run functional enrichment analysis. This list consisted of 571 upregulated and 135 downregulated genes (Tables S4 and S5). GO enrichment analysis was used to study biological processes, cellular components, and molecular functions implicated in IRI. The top 20 up- and down-regulated terms are reported in Figure 1 and Figure 2, respectively. Among the most highly enriched biological processes, several upregulated DEGs related to immune signaling and cell death regulation, while downregulated DEGs were largely involved in intercellular signaling and synaptic transmission. The upregulated genes related to molecular functions primarily involved binding proteins, while downregulated genes related to transport channels. Finally, among enriched cellular component DEGs, those upregulated were found to relate to the cell periphery (GO:0071944), while those composing the synapse (GO:0045202) were downregulated.

**Figure 3:**
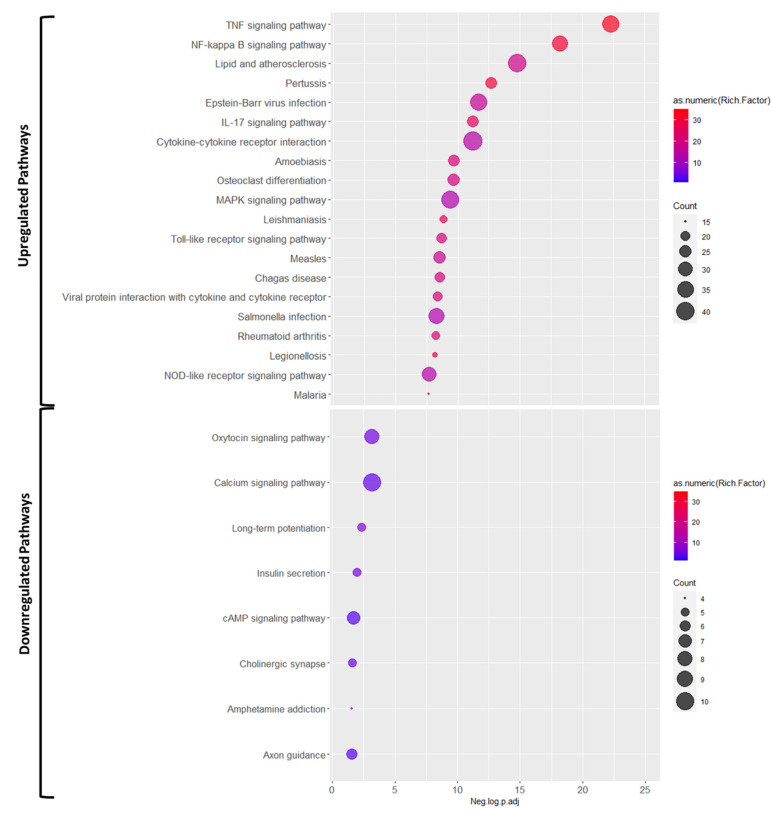
The results of KEGG enrichment pathway for upregulated and downregulated DEGs for IR condition (list of all significant terms can be found in Tables S12 and S13).

Further, we studied the engaged pathways using KEGG enrichment analysis (Figure 3). The pathway analysis for upregulated genes highlighted multiple inflammatory response pathways, such as TNF, NF-κB, and IL-12 signaling pathways, as well as cytokine-cytokine receptor interaction, and toll-like receptor signaling. Conversely, the top dysregulated pathways involving significantly downregulated genes included oxytocin and calcium signaling pathways and long-term potentiation.

### 3.3. Organ-Specific Differential Expression in Response to IRI

**Figure 4:**
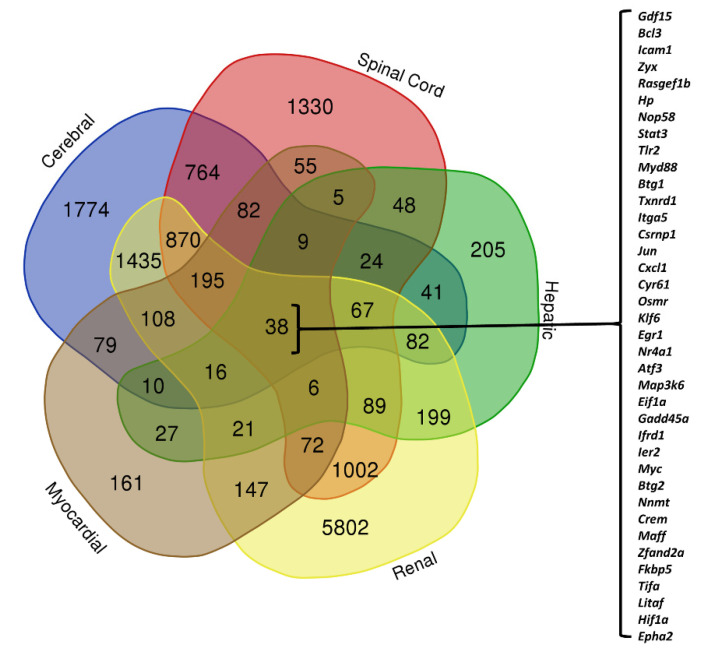
Venn diagram of all included DEGs categorized based on source organ. The values represent the number of DEGs in each division (details of diagram in Table S2).

To illustrate transcriptomic changes in each organ, we used a Venn diagram of DEGs from the selected studies (see Table 1) categorized by organ (details are provided in Table S2). We found 38 DEGs common across all five organs (Figure 4). Among these were *Bcl3, Icam1, Itag5, Atf3,* and *Myc*, which are involved in apoptosis and cell death. We also observed transcriptomic changes in genes involved in the inflammatory response, including *Stat3, Tlr2, Cxcl1, Epha2,* and *Myd88.* Furthermore, based on these 38 genes, TNF, NF-κB, and MAPK signaling pathways appeared to be highly implicated in IRI response.

## 4. Discussion

### 4.1. Therapeutic Interventions Act on Genes and Pathways Involved in IRI

Therapeutic interventions were investigated for their efficacy at attenuating IRI. These have included ischemic pre- and post-conditioning and delivery of stem cells to the affected organs. It is likely that treatment approaches that achieve beneficial effects act on the same genes and pathways implicated in IRI via their counter-regulation. Here, we will compare RNAseq data from therapeutic interventions with the results of our cross-comparison of DEGs and pathways across IRI.

### 4.2. Ischemic Postconditioning

Zhang et al. (2019) used ischemic postconditioning (IPO) of the liver in a wildtype mouse model, whereby, following 1 h of ischemia, the porta hepatis was re-perfused for three 5 s cycles prior to full reperfusion for four hours [18]. The extent of injury was then compared between animals that received IPO and those that only underwent IRI. Among those we found to be implicated across IRI conditions, the authors found *Cyr61* to be downregulated by IPO. *Cyr61* belongs to the CCN protein family and regulates cell adhesion, proliferation, and apoptosis [36,37]. Additionally, they observed significant upregulation of *Atf3* in IPO, the product of which is a member of the ATF/cAMP-responsive element binding protein transcription factor family and has been found to repress macrophage pro-inflammatory gene expression in IRI [38,39]. Several pathways the authors found to be differentially regulated in liver IPO were similar to those we found to be highly implicated across IRI studies. IPO significantly downregulated the MAPK and IL-17 signaling pathways, which modulate cell proliferation and apoptosis, and inflammatory cytokines and apoptosis, respectively, suggesting their role as potential protective mechanisms in IRI [18].

### 4.3. Caloric and Hypoxic Preconditioning

Additional conditioning methods, including caloric restriction (CR) and hypoxic preconditioning (HP), were investigated for their efficacy in attenuating renal IRI in wildtype mice [20]. Animals were subjected to either a 30% reduction in food consumption per week or three days of hypoxic exposure for 2, 4, and 8 h sequentially, prior to undergoing 40 min clamping of the left renal pedicle followed by 4, 24, or 72 h of reperfusion. CR was found to downregulate apoptotic regulator genes *Bik* and *Dynll1* that were inversely upregulated in IRI. In non-preconditioned animals, IRI was found to upregulate similar pro-inflammatory pathways to those enriched in our KEGG analysis, including NF-κB, TNF, and IL-17. Both HP and CR were found to regulate several genes overlapping with IRI in an inverse manner, providing significant protective effects [20].

### 4.4. Stem Cell Therapy Approaches

Stem cell treatment was also employed to aid in reparative processes and supplement depleted cell populations in order to encourage functional recovery. Using a rodent middle cerebral artery occlusion (MCAO) model of stroke, pluripotent hematopoietic stem/progenitor cells (HSPC) were delivered 24 h post-reperfusion and were successful in reducing infarct volume and improving neurologic score [29]. HSPC treatment was found to upregulate IL-10 expression, suggesting anti-inflammatory protection by attenuating immune cell recruitment. This coincides with our findings of upregulation of pro-inflammatory IL-10 signaling in IRI. RNA sequencing also identified significantly increased metallothionein (MT-1) in transplanted cells, a cysteine-rich, free radical binding-protein that is protective in oxidative stress [40]. Further, multiple genes involved in the pro-inflammatory DENN/ERK/MAPK pathway were found to be significantly upregulated in IRI, adding to the DEGs we found to be highly implicated in the MAPK pathway [29].

Culturing methods were compared to determine the efficacy of spheroid 3D cultures, relative to 2D, for facilitating engraftment following transplant. Multipotent human umbilical cord-derived mesenchymal stem cells (UC-MSC) were employed as a protective treatment in a rat model of hepatic IRI, using 2D and 3D cultured cells, as well as untreated controls, in order to determine an optimal treatment approach [30]. Both treatment conditions were found to reduce mRNA expression of pro-inflammatory TNF, and upregulate the expression of potentially protective IL-6 relative to the untreated controls, recapitulating the protective effects of targeting DEGs most highly implicated in IRI. Three-dimensional-cultured MSCs were found to be more effective at attenuating inflammation by acting on the TNF signaling pathway, as illustrated by their upregulation of TNF-α-induced protein 8, a potent inhibitor of apoptosis [41], and TNF-α-stimulated gene-6, a modulator of macrophage polarization [42]. Further, 3D cultured MSC were found to differentially regulate trophic factors, such as vascular endothelial growth factor, hepatocyte growth factor, and basic fibroblast growth factor, potentially affecting their subsequent engraftment and suggesting a therapeutic target to improve stem cell treatment efficacy [30].

## 5. Conclusions

In summary, IRI inflicts extensive damage by inhibiting vascular circulation, thereby disrupting metabolic and ionic regulation. This is then followed by the restoration of blood flow across oxygen-depleted areas, often overwhelming membrane flux and causing the formation of free radicals. This results in the initiation of several inflammatory kinase pathways as cells respond to ROS-induced damage and attempt to reinstate homeostasis. Several pathways are canonically dysregulated across IRI modalities, with immune signaling and cell death regulating pathways being the primary targets. Those modulating metabolic processes, ionic exchange, and developmental pathways are also highly implicated, as illustrated by functional clusters of genes displaying significant differential expression in ischemia-reperfusion injury models.

Our analysis of transcriptomic changes in IRI across organs was limited by a lack of studies providing comprehensive data on differential gene expression in IRI. Understanding the roles of these genes and pathways at large is a critical step in advancing therapeutic strategies, and the advent of RNA sequencing provides unprecedented insight into these dynamics. In this study, we analyzed differential gene expression and pathway regulation across existing studies to provide a broad overview of genes canonically implicated in IRI. Future research may apply these methods to create a more detailed, comprehensive list of transcriptomic changes in response to ischemia-reperfusion. This information may be used to guide research and optimize therapeutic approaches going forward.

## Figures and Tables

**Table 1:** Summary of studied articles in this study.

References	Reperfusion Model	Species	Reperfusion Duration
Zhou et al. 2020 [15] *	Spinal cord IRI	Sprague-Dawley rat	48 h
Cai et al. 2019 [16] *	Cerebral IRI	C57BL/6J mice	1, 3, 7, 14, 28 d
Dergunova et al. 2018 [17] *	Cerebral IRI	Wistar rats	4.5, 24 h
Zhang et al. 2019 [18] *	Hepatic IRI	C57BL/6J mice	4 h
Shi et al. 2017 [19] *	Cerebral IRI	Sprague-Dawley rat	45 min, 6 h, 12 h and 18 h
Johnsen et al. 2020 [20] *	Renal IRI	C57BL/6J mice	4, 24 h
Li et al. 2020 [21] *	Myocardial IRI	C57BL/6J mice	40 min
Mo et al. 2019 [22]	Myocardial IRI	C57BL/6J mice	1 h
Kestner et al. 2020 [23]	Cerebral IRI	C57BL/6J mice	24 h
Kolling et al. 2018 [24]	Renal IRI	C57BL/6J mice	24 h
Wang et al. 2017 [25]	Cerebral IRI	Wistar rats	24 h
Park et al. 2020 [26]	Renal IRI	Human	10 min
Giraud et al. 2018 [27]	Renal IRI	Porcine	6, 24 h
Deng et al. 2020 [28]	Myocardial IRI	C57BL/6J mice	24 h
Smith et al. 2018 [29]	Cerebral IRI	C57BL/6J mice	24 h, 48 h, 1 w, 2 w
Sun et al. 2018 [30]	Hepatic IRI	Sprague-Dawley rats	6, 24, 48 h
Liu et al. 2017 [31]	Renal IRI	C57BL/6J mice	2 h, 4 h, 1 d, 2 d, 3 d, 1 w, 2 w, 4 w, 6 months, 12 months

* The RNA-seq data from these articles were used in the comparison analysis.

**Table 2:** Highly repeated up-regulated genes in IRI.

Upregulated	Comments
*Itga5*	Programmed cell death
*Atf3*	Cell proliferation, programmed cell death
*Cyr61*	Cell proliferation, programmed cell death
*Litaf*	Programmed cell death
*Stat3*	Programmed cell death, inflammatory response
*Icam1*	Inflammatory response, programmed cell death
*Sik1*	Programmed cell death
*Tlr2*	Inflammatory response
*Zc3h12a*	Inflammatory response
*Epha2*	Inflammatory response
*Maff*	Response to stress
*Nes*	Cell proliferation, programmed cell death
*Hmox1*	Response to stress, inflammatory response
*Myc*	Programmed cell death, response to stress
*Cd44*	Inflammatory response
*Hbegf*	Cell proliferation
*Ifrd1*	Developmental process
*Pdpn*	Cell proliferation, programmed cell death
*Cd14*	Inflammatory response
*Jun*	Cell proliferation, programmed cell death
*Csf1*	Inflammatory response
*Gadd45a*	Programmed cell death, response to stress
*Ccl2*	Inflammatory response
*Klf6*	Developmental process
*Hspb1*	Response to stress, programmed cell death
*Olr1*	Inflammatory response
*Mafk*	Response to stress
*Zyx*	Inflammatory response
*Osmr*	Inflammatory response
*Tifa*	Response to stress
*Bcl3*	Inflammatory response, programmed cell death
*Irf1*	Cell proliferation, programmed cell death
*Tnfrsf1a*	Inflammatory response
*Cxcl1*	Inflammatory response
*Txnrd1*	Cell proliferation, response to stress
*Spon2*	Cell surface adhesion and signaling, response to stress
*Myd88*	Inflammatory response
*Bach1*	Response to stress

**Table 3:** Highly repeated down-regulated genes in IRI.

Downregulated	Comments
*Cabp1*	Voltage-gated calcium ion channel regulator
*Pclo*	Presynaptic cytoskeletal matrix
*Npy1r*	Synaptic signaling
*Tenm2*	Formation of growth cone in neural cells
*Cbx7*	Cellular lifespan regulator
*Camk4*	Transcriptional regulation
*Kcng1*	Voltage-gated potassium (Kv) channels subunit
*L1cam*	Cell surface adhesion and signaling
*Dlg2*	Response to oxidative stress and DNA damage
*Scn4b*	Sodium channel beta subunit
*Kcnab1*	Cytoplasmic potassium channel subunit
*Rasgrp1*	T cell/B cell regulator
*Kcnt1*	Potassium-sodium activated channel subunit
*Adora2a*	Cell surface adhesion and signaling
*Phactr1*	Endothelial cell survival
*Lzts3*	Maturation of dendritic spines
*Stard8*	Cell surface adhesion and signaling
*Agrn*	Cytoplasmic Ca ion regulator
*Cacnb3*	Voltage-dependent calcium channel regulatory subunit
*Cobl*	Reorganization of the actin cytoskeleton

## Data Availability

The data presented in this study are available in the supplementary material of this article.

## Supplementary Materials

The following are available online at https://www.mdpi.com/article/10.3390/cells10071838/s1, Table S1: The full list of DEGs reported in each publication, Table S2: The detailed results of Venn diagram, Table S3: The comparison analysis of all DEGs reported in the analysed papers and number of time they were repeated, Table S4: The full list of gene’s that were consistently reported to get upregulated in IRI (at least 4 time reported by papers), Table S5: The full list of gene’s that were consistently reported to get downregulated in IRI (at least 4 time reported by papers), Table S6: The list of enriched GO terms in Biological processes domain for Upregulated genes, Table S7: The list of enriched GO terms in Cellular Component domain for Upregulated genes, Table S8: The list of enriched GO terms in Molecular Function domain for Upregulated genes, Table S9: The list of enriched GO terms in Biological processes domain for Downregulated genes, Table S10: The list of enriched GO terms in Cellular Component domain for Downregulated genes, Table S11: The list of enriched GO terms in Molecular Function domain for Downregulated genes, Table S12: The list of enriched KEGG pathways for Upregulated genes, Table S13: The list of enriched KEGG pathways for Downregulated genes.

## Abbreviations

**Abbreviation**	**Explanation**
ATF	activating transcription factors
BP	biological process
CaMK	calcium/calmodulin-dependent protein kinase
cAMP	cyclic adenosine monophosphate
CCN	CYR61 (cysteine-rich angiogenic protein 61), CTGF (connective tissue growth factor), NOV (nephroblastoma overexpressed)
CC	cellular components
CR	caloric restriction
DEGs	differentially expressed genes
DENN	differentially expressed in normal and neoplastic cells
ERK	extracellular signal-regulated kinases
FDR	false discovery rate
GO	Gene Ontology
HP	hypoxic preconditioning
HSPC	pluripotent hematopoietic stem/progenitor cells
IL-10	Interleukin-10
IL-12	Interleukin-12
IL-17	Interleukin-17
IL-6	Interleukin-6
IPO	ischemic postconditioning
IRI	ischemia reperfusion injury
KEGG	Kyoto Encyclopedia of Genes and Genomes
MAPK	mitogen-activated protein kinase
MCAO	middle cerebral artery occlusion
MF	molecular function
MSC	mesenchymal stem cells
MT-1	metallothionein
NF-κB	nuclear factor kappa-light-chain-enhancer of activated B cells
PANTHER	protein analysis through evolutionary relationships
ROS	reactive oxidative species
TNF	tumor necrosis factor alpha
UC-MSC	umbilical cord-derived mesenchymal stem cells
